# Identification of LSM family members as potential chemoresistance predictive and therapeutic biomarkers for gastric cancer

**DOI:** 10.3389/fonc.2023.1119945

**Published:** 2023-03-17

**Authors:** Qianhui Liu, Qinghai Lian, Yingqiu Song, Shangbin Yang, Changchang Jia, Jiafeng Fang

**Affiliations:** ^1^ Department of Gastrointestinal Surgery, The Third Affiliated Hospital of Sun Yat-sen University, Guangzhou, China; ^2^ Department of Cell-Gene Therapy Translational Medicine Research Center, The Third Affiliated Hospital of Sun Yat-Sen University, Guangzhou, China

**Keywords:** LSM family, gastric cancer, 5-fluorouracil, chemotherapy resistance, immune infiltration, prognosis

## Abstract

**Introduction:**

The Like-Smith (LSM) family plays a critical role in the progression of several cancers. However, the function of LSMs in chemoresistance of gastric cancer (GC) is still elusive.

**Methods:**

The Cancer Genome Atlas (TCGA) database, Gene Expression Omnibus (GEO) database and Tumor Immune Estimation Resource Analysis (TIMER) were utilized to analyze the expression, prognostic value and immune infiltration of LSMs in GC patients. Moreover, qPCR and immunohistochemistry (IHC) experiment were conducted with clinical samples.

**Results:**

The expression of LSMs was upregulated in GC tissues and most of LSMs were negatively correlated with overall survival of GC patients with 5-fluorouracil (5-FU) treatment. We further revealed that LSM5, 7 and 8 were hub genes of GEO (GSE14210). Besides, the qPCR results demonstrated that a higher level of LSM5 and LSM8 was associated with 5-FU chemoresistance in GC. Moreover, both TIMER and IHC revealed that a lower expression of LSM5 and LSM8 was correlated with high infiltration of T cells, regulatory T cells, B cells, macrophages, and neutrophils.

**Discussion:**

Our study systematically investigated the expression pattern and biological features of LSM family members in GC, and identified LSM5 and LSM8 as potential biomarkers in GC with 5-FU chemotherapy.

## 1 Introduction

Globally, gastric cancer (GC) is the fifth most common cancer and ranks third in terms of cancerrelated death (1). Currently, chemotherapy has been one of the basic therapies for GC, and 5fluorouracil (5-Fu), together with its derivatives, is a cornerstone of standard chemotherapy regimens (2). However, poor clinical outcome exists in GC due to the rapid emergence of chemotherapy resistance, which has become a major hurdle for GC therapy. Although there is a great development of combinining targeted therapy or immunotherapy for the treatment of GC these decades (3), their therapeutic effect is still limited and many obstacles remain. Therefore, identifying new prognostic biomarker and improving chemotherapy sensitivity are in urgent need.

Smith-like (LSM) proteins are known as a family of RNA-binding proteins that appear in all essential cellular organisms (4). It consists of 13 members (e.g. LSM1, LSM2, LSM3, LSM4, LSM5, LSM6, LSM7, LSM8, LSM10, LSM11, LSM12, LSM14A, and LSM14B), which are generally involved in various cellular biological processes (e.g. RNA-processing tasks and ion mobilizations) (5). For example, the LSM2-8 complex in the nucleus functions in pre-mRNA splicing by interacting with the U6 snRNA (6). Moreover, the oncologic roles of LSM family members have also been identified in several tumor types. Particularly, the LSM1 protein plays a role in the cellular conversion and progression of breast cancer (7), and pancreatic adenocarcinoma (8). Additionally, LSM8 had a strong relationship with the development of Hashimoto’s thyroiditis (9). Furthemore, a previous study by Zhu et al. also identified a novel LSM8-MET that could potentially act as an additional tertiary resistance mechanism (10), and E C Little et al. found that inducing LSM1 expression resulted in decreased chemotherapeutic sensitivity of pancreatic adenocarcinoma cells (8). Although there are few reports of LSM family members in tumor chemoresistance, it makes us more interested to explore the underlying function of LSMs as a whole in chemotherapy and progression of GC.

Through public databases and multiple bioinformatics analysis, we comprehensively investigated the role of LSM family genes in tumor microenvironment and the prediction value for 5-FU treatments in GC. Additionally, with the analysis of tumor immunity and chemoresistance, we were the first to demonstrate that LSM5 and LSM8 were tightly correlated with chemoresistance and immune infiltration, and thus might be potential biomarkers for predictions of survival in GC patients.

## 2 Materials and methods

### 2.1 Data acquisition

The Cancer Genome Atlas (TCGA) database (https://portal.gdc.cancer.gov/) and the GenotypeTissue Expression (GTEx) database were utilized to obtain the RNA-seq data and relevant clinical data across 33 tumor types and normal tissues of 15,776 samples. We also collected 375 STAD patients accompanied with 32 normal tissues from the TCGA database. Then, we transferred RNAseq data in FPKM format to TPM format and further analyzed expression pattern for LSM family members. GSE14210 dataset was obtained from the Gene Expression Omnibus (GEO, https://www.ncbi.nlm.nih.gov/geo/) for validation.

### 2.2 cBioPortal

cBioPortal (www.cbioportal.org) is a web resource that allows for the exploration, visualization, and analysis of multidimensional cancer genomics data (11). We analyzed the genomic profiles of 13 members of the LSM family to visualize the full details of each type of mutation in each individual sample.

### 2.3 Gene expression profiling interactive analysis 2

GEPIA2 (http://gepia2.cancer-pku.cn/#index) is a multidimensional cancer genome dataset that integrates large amounts of data from The Cancer Genome Atlas (TCGA) and the Genotype-Tissue Expression Project (12). Here, the correlation between LSM and clinical stage was evaluated, and the module “Similar Gene Detection” was used to identify the most similar 100 genes for LSM5, 7, 8.

### 2.4 DNA Methylation

Methsurv (https://biit.cs.ut.ee/methsurv/) was used to evaluate the methylation level (13). Here we analyzed the CpG sites of the LSM family members in the STAD samples.

### 2.5 Differentially expressed gene analysis

Based on the median expression levels of LSM5, LSM7 and LSM8, the expression data (HTseqCounts) were divided into high and low expression groups and further analyzed by unpaired Student’ s t-test within the DESeq2 R package (3.6.3). Adjusted p <0.05 and |log2-fold change (FC)|>1.5 were considered as thresholds for the DEGs.

### 2.6 Tumor immune estimation resource analysis

TIMER (cistrome.shinyapps.io/timer) is a public tool to comprehensively investigate tumor-immune interactions (14). In this work, “Gene module” and scatterplots were obtained to analyze the correlation between the expression level of LSM members and the infiltrating of diverse immune cells in STAD.

### 2.7 Survival analysis

We determined the correlations between LSMs mRNA expression levels and the survival of GC patients using the KM Plotter (http://www.kmplot.com) (accessed on 18 December 2021) databases (15). This online public database is a robust platform for visualizing patients’ survival across several cancer types. Overall survival (OS) in GC based on 5-FU chemotherapy for the LSM gene family was set as the default in the KM-plot database.

### 2.8 Protein-protein interaction analysis

The online STRING database (https://string-db.org/, V11.0) (accessed on 18 December 2021) was used to analyze all publicly available sources of information and predict protein-protein interactions in the organism (16). The STRING analysis data were imported using Cytoscape software (version 3.8.1). In this work, a network compose of LSM family and their 50 similar neighboring genes was constructed using a protein-protein interaction module.

### 2.9 The receiver operating characteristic

ROC analysis of LSMs were realized by the pROC package. The calculated area under the curve (AUC) value ranges, which were from 0.5 to 1.0, indicated the discrimination ability of 50%–100%.

### 2.10 Enrichment analysis

With the selection of 100 similar expression genes for LSM5 and LSM8 through the GEPIA2 dataset, the “org.Hs.eg.db” (v3.10.0) R package was used to convert entrez ID to the gene symbol. The “ClusterProfiler” (v3.14.3) R package was used for the functional annotation. GO analysis includes cellular component (CC), molecular function (MF), and biological process (BP).

### 2.11 Patients and tissue samples

Thirteen GC patients with neoadjuvant chemotherapy treated in the Third Affiliated Hospital of Sun Yat-Sun University between January 2013 and December 2021 were enrolled in this study. Fresh frozen normal stomach and tumor tissues were obtained. All patients enrolled in this study had provided the written informed consent. Experiments related to human or human samples were approved by the Ethics Committee of Third Affiliated Hospital of Sun Yat-Sun University ([2022]02-169-01). The effects of patients with 5-FU chemotherapy were evaluated according to RECIST(Response Evaluation Criteria In Solid Tumours)1.1 (17), including CR (complete response), PR (partial response), SD (stable disease) and PD (progressive disease). We then divided the patients into two groups, sensitive (CR, PR) or non-sensitive (SD, PD).

### 2.12 RNA extraction and qRT-PCR

Total RNA was extracted with TRIZOL reagent (Thermo Fisher, USA). The cDNA was prepared using HifairTM II 1st Strand cDNA Synthesis SuperMix Kit (YEASEN, Shanghai, China) according to the manufacturer’s protocol. Real time q-PCR was performed using ChamQ SYBR qPCR Master Mix (Vazyme, Nanjing, China) following instructions. 18S(Human) was used as an internal reference to normalize the expression of other mRNAs. Sequences used in this study were listed as follows:

LSM5 forward (F): 5′- TGGTACTGGAAGATGTCACTGAG-3′, LSM5 reverse (R): 5′CACACTTCAGGTCCTTCTCCTC-3′; LSM7 (F): 5′- CACTCCTCAACCTTGTGCTGGA -3′,
LSM7 (R): 5′- GCAGATTAGCACCACGGACGTG -3′; LSM8 (F): 5′-
CGAGTATTCAGCTCTTCACAGGG -3′, LSM8 (R): 5′- CCCAAATCAAGCGCAGAATCTGT -3′; 18S (F): 5′- ACCCGTTGAACCCCATTCGTGA -3′, 18S (R): 5′GCCTCACTAAACCATCCAATCGG -3′.

### 2.13 Immunohistochemistry

Nine matched GC tissues and the adjacent normal tissues were selected from the 13 GC patients with LSM5, LSM8 high or low expression. After formalin fixed and paraffin embedded, the tissues were cut into 5 µm slides and then received IHC staining. Briefly, the slides were dewaxed with xylene and ethanol, and antigen repaired with EDTA buffer (pH 8.0) in a microwave oven. After being incubated with 5% goat serum diluted with TBS buffer containing 1% Tween-20, the slides were respectively probed with the primary antibody against CD3 (1:400), CD8 (1:1000), FoxP3 (1:400), CD68 (1:500), CD20 (1:500) and CD66b (1:500) overnight at 4°C and the corresponding second antibody for 1 hour at room temperature, and the DAB color development time of these immune cell markers was 3 minutes. To analyze the expression level of T cells, CD8+ T cells, regulatory T cells (Tregs), macrophage, B cells and neutrophilia in different gastric tissue samples, we counted the cells to compare the differences between chemotherapy sensitive and non-sensitive.

### 2.14 Statistical analysis

Statistical analyses were performed using GraphPad Prism (GraphPad, version 7). The statistical data of TCGA datasets were processed by R 3.6.3. Wilcoxon rank-sum test and Wilcoxon signed-rank test were applied for comparing the expression of LSMs between GC and the normal group. Chi-square test and t-test were applied for variance analysis, and Spearman rank correlation method was for correlation analysis. Each of the statistical tests was two- tailed, and a *P*-value <0.05 was identified statistically significant.

## 3 Results

### 3.1 The expression level of LSMs significantly elevated in multiple cancers including gastric cancer

**Figure 1:**
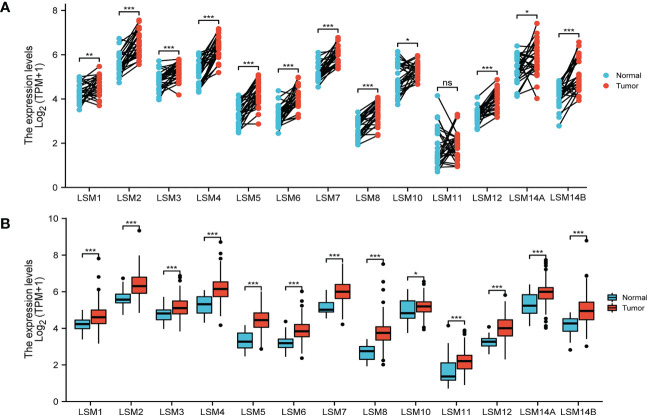
Expression level of 13 LSM members significantly up-regulated in GC by TCGA database analysis. **(A)** The expression level of LSM family members in gastric cancer tissues and their paired normal tissues. **(B)** The expression level of LSM family members in gastric cancer tissues and their unmatched normal tissues. TCGA database (https://portal.gdc.cancer.gov/); ns, p ≥ 0.05; *p < 0.05; **p < 0.01; *** p < 0.001.

The transcriptional level of LSM family members was analyzed in 33 cancer datasets from the TCGA database. LSMs was up-regulated in most cancer tissues out of the 33 and all LSM members were significantly higher in GC tissues compared to the normal tissues (**Figure S1**). Using the GEPIA dataset, we compared the mRNA expression of LSMs between GC and normal tissues. The results indicated that the expression levels of all LSM members except LSM11 elevated in GC tissues than their matched normal tissues (**Figure 1A**). Also, the comparison in unpaired GC tissues showed that expression of LSMs was greatly higher in GC tissues (**Figure 1B**), which was consistent with those from TCGA.

### 3.2 Relationship between the mRNA Levels of LSMs and the clinicopathological parameters of patients with gastric cancer

We then assessed the correlation between the expression of LSMs and the pathological stage of GC patients. All LSM members except LSM10 in GC patients with any pathological stage were markedly different from normal tissues. These data also showed that the expression of LSM1 was different between stage I and stage II, while that of LSM8 differed from stage II and stage III (**Figure S2**).

### 3.3 Genetic alteration, correlation, and interaction analyses of LSMs in GC patients

**Figure 2:**
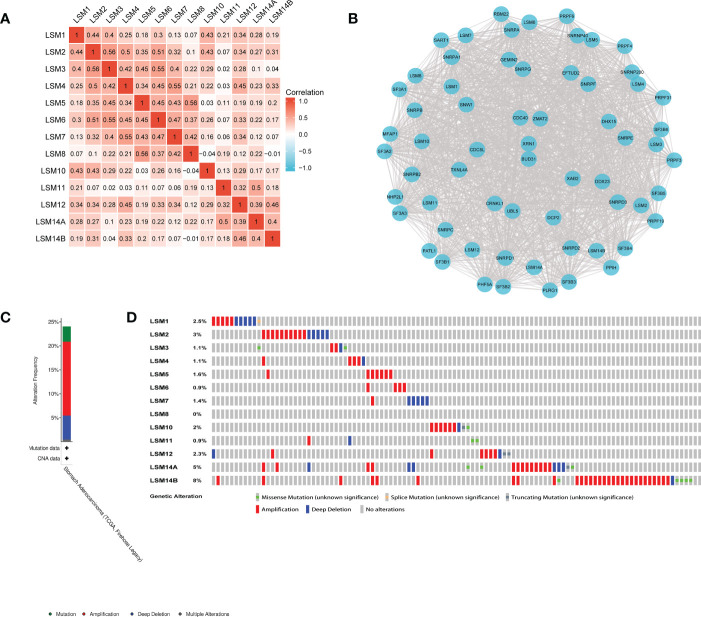
Heatmap, network and genetic mutations of LSM proteins. **(A)** the correlation among the LSM members by the Pearson correlation analysis from TCGA database. **(B)** Network comprising LSM1-14B and their most closely associated genes by STRING and Cytoscape analysis. **(C, D)** Genetic mutation analysis of LSM proteins by cBioPortal.

We examined the correlation among the LSM members using the Pearson correlation analysis. Significantly positive correlations were observed between LSM1 and 2, 3, 10, 12; LSM2 and 3, 4, 6, 10; LSM3 and 5, 6, while negative correlation was observed between LSM8 and 10, 14B. Overall, the topmost Pearson coefficient (0.56) was observed between LSM2 and 3, LSM5 and 8 (**Figure 2A**). Moreover, we conducted a protein-protein interaction (PPI) network analysis of the differentially expressed LSMs with Cytoscape to explore the potential interactions among them (**Figure 2B**). Also, we analyzed the genetic alterations of LSMs in GC patients by using the cBioPortal online tool. Overall, two or more alterations were detected in different subtypes of GC, and amplification alterations were more common in GC (**Figures 2C, D**).

### 3.4 DNA methylation analysis of LSMs

**Figure 3:**
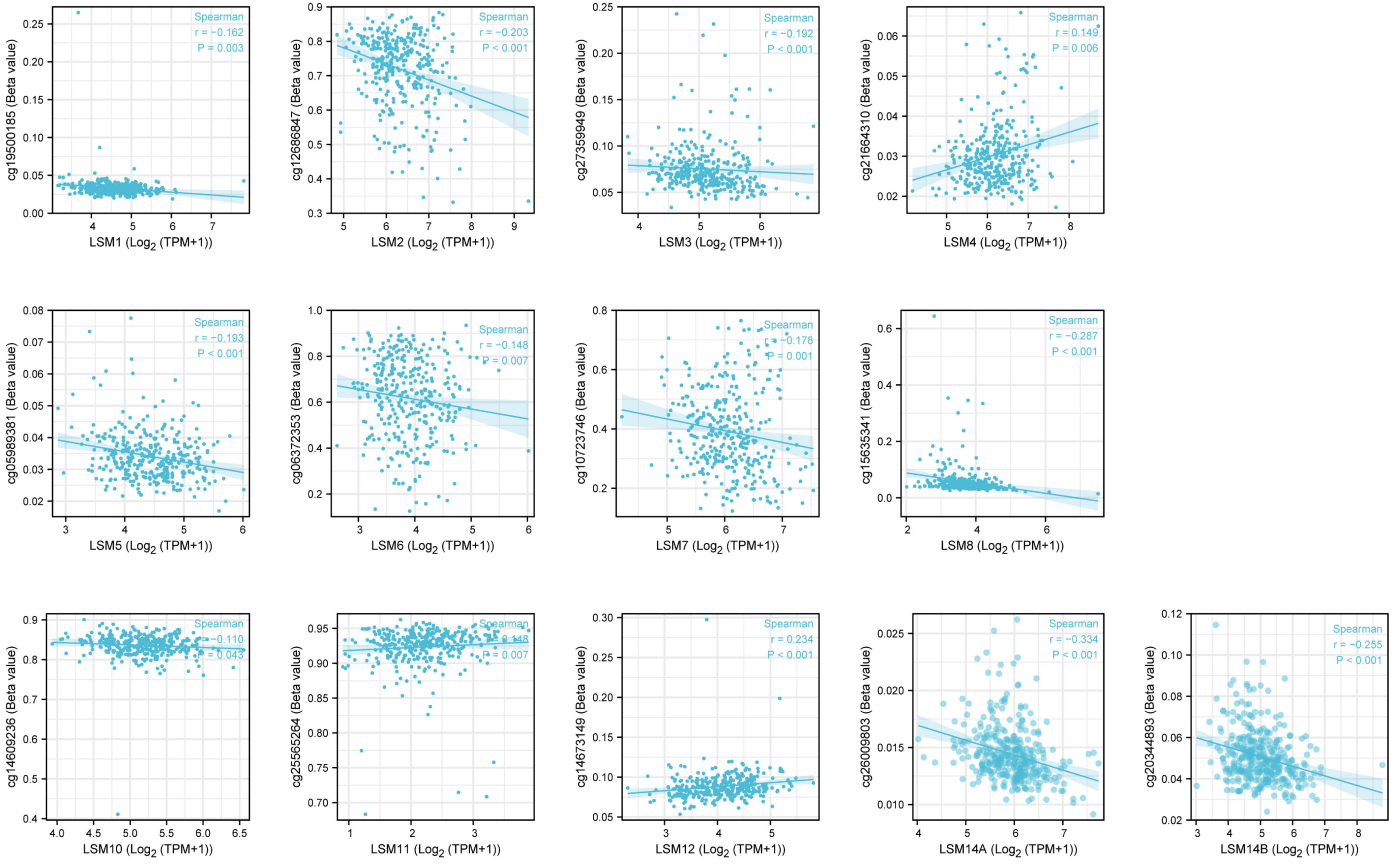
The DNA methylation level of LSM family members in GC by Methsurv analysis. From TCGA RNAseq data in level 3 HTSeq-FPKM format and ilumina human methylation 450 methylation data in STAD (gastric cancer) project.

DNA methylation is frequent epigenetic event that relates to the viability of cancer cells, which gains significant resistance to anticancer drugs and escapes programmed cell death. Here, we investigated that LSM1, 2, 3, 5, 6, 7, 8, 10, 14A, 14B were negatively correlated with DNA methylation, while LSM4, 11, 12 showed positive correlations with it (**Figure 3**).

### 3.5 Prognostic value of the mRNA expression of LSMs for GC patients undergoing 5-FU based chemotherapy

**Figure 4:**
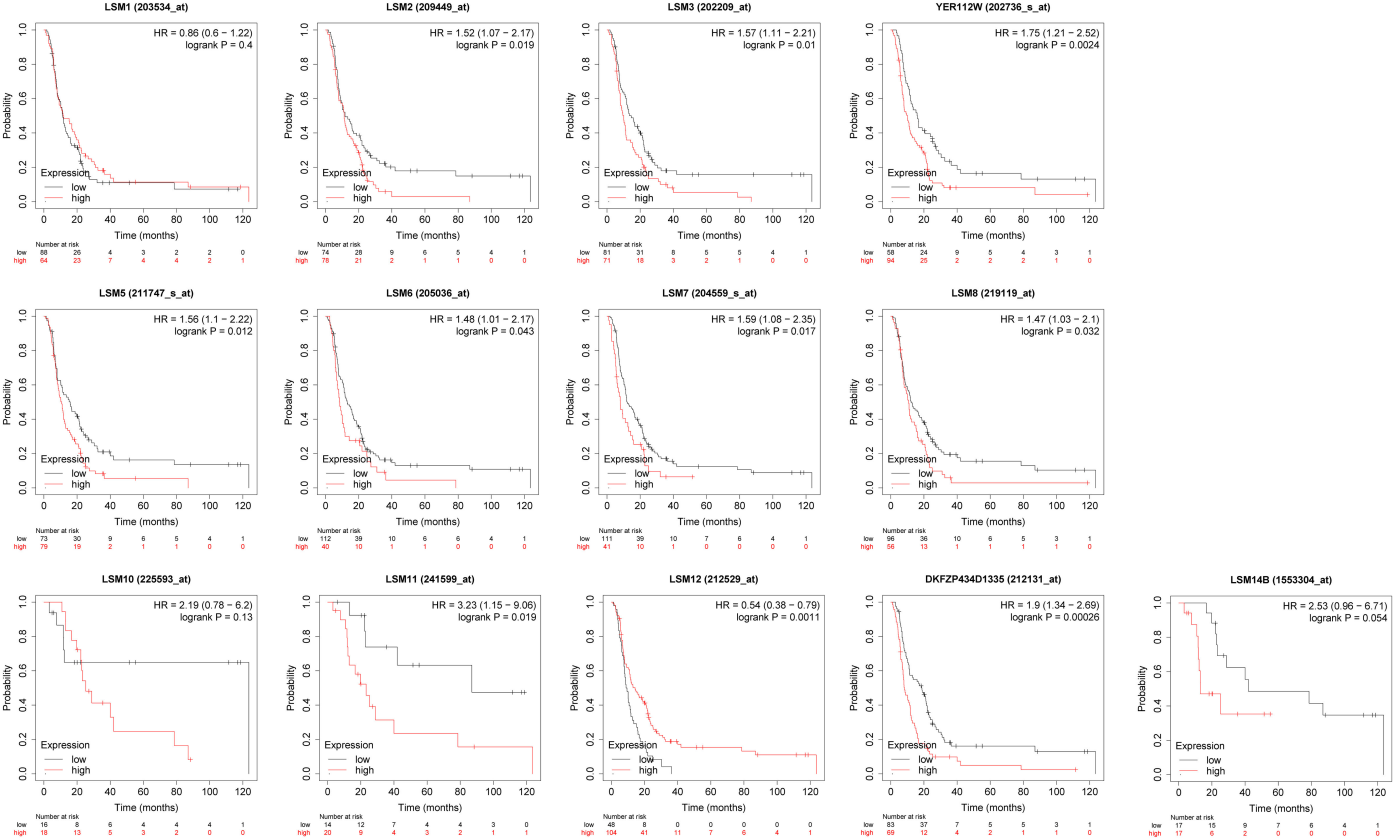
Prognostic value of LSMs in GC by Kaplan-Meier plotter. 9 of 13 members of the LSM family except LSM1, LSM10 and LSM14B were significantly associated with OS of GC patients with 5-FU chemotherapy from Kaplan-Meier Plotter (https://kmplot.com/analysis/).

We assessed the associations between the mRNA expression of LSM family members and OS using KM survival analysis in GC patients with 5-FU chemotherapy. As shown in **Figure 4**, the results indicated that 9 of 13 members of the LSM family were significantly associated with poor OS of GC patients undergoing 5-FU based chemotherapy, such as LSM2 (HR = 1.52, p for trend = 0.019), LSM3 (HR = 1.57, p for trend = 0.01), LSM4 (HR = 1.75, p for trend = 0.0024), LSM5 (HR = 1.56, p for trend = 0.012), LSM6 (HR = 1.48, p for trend = 0.043), LSM7 (HR = 1.59, p for trend = 0.017), LSM8 (HR = 1.47, p for trend = 0.032), LSM11(HR = 3.23, p for trend = 0.019), and LSM14A (HR= 1.9, p for trend = 0.00026). In contrast, high expression levels of LSM12 indicated longer OS (HR = 0.54, p for trend = 0.0011). In addition, LSM1, LSM10 and LSM14B showed non-significant prognostic values.

### 3.6 Identification of LSM5 and LSM8 as unfavorable biomarkers related to 5-FU chemotherapy sensitivity

**Figure 5:**
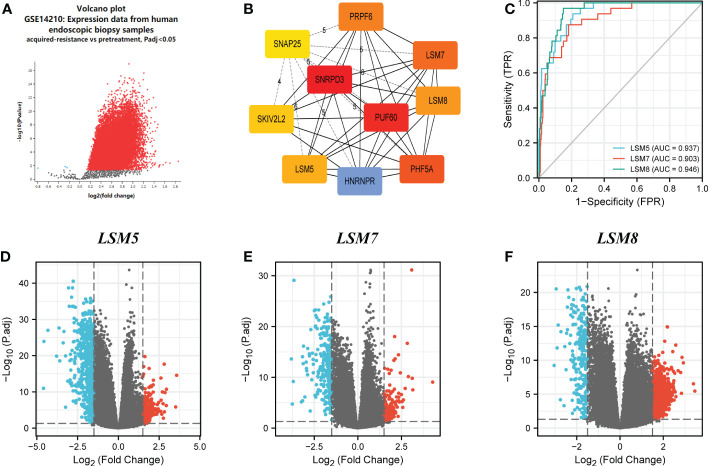
Differential expression analysis and the predictive value of LSM5, LSM7 and LSM8 in GC. **(A)** Volcano plots of the differentially expressed genes (DEGs) screened by GEO2R in GSE14210 from GEO database. **(B)** the top 10 hub genes of above upregulated DEGs by MCC degree from STRING and Cytoscape. **(C)** Receiver operating characteristic (ROC) curve for LSM5, LSM7 and LSM8 expression in GC by the pROC package by the pROC package. **(D–F)** Volcano plots of DEGs of LSM5, LSM7 and LSM8 in GC from TCGA database.

GSE14210 dataset has gene expression data of pretreatment and posttreatment endoscopic biopsy samples collected from cisplatin and fluorouracil combination chemotherapy in GC patients. Through GEO2R, we found 147 different expression genes as shown by volcano plot (**Figure 5A**), and analysis of 10 hub genes revealed the existence of LSM5, 7 and 8 (**Figure 5B**). Moreover, the AUC values of LSM5, 7 and 8 in the TCGA GC cohort were 0.937, 0.903, and 0.946 respectively, which were interestingly higher than the AUC values of the rest LSM members (**Figures 5C**; **S3A**). And a time-ROC curve of these three members showed relatively high prognostic ability (**Figure S3B**). In summary, LSM5, 7 and 8 were considered primarily correlated with 5-FU chemoresistance in GC.

**Figure 6:**
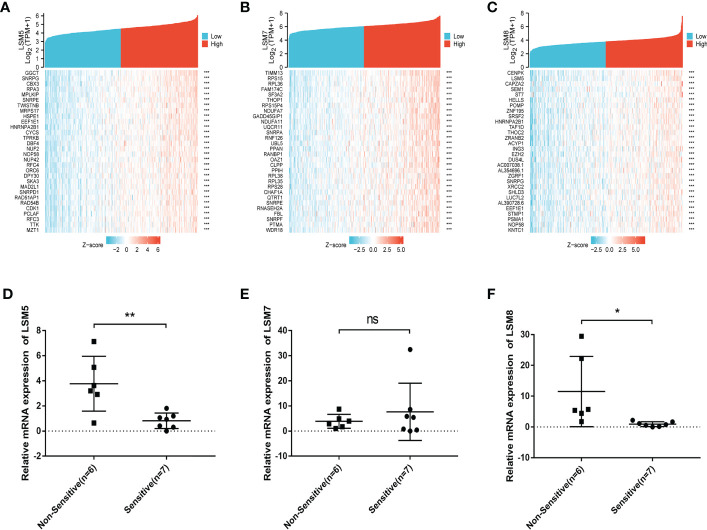
The positively correlated 50 genes and mRNA expression levels of LSM5, LSM7 and LSM8 in GC. **(A-C)** The gene co-expression heatmap of the top 50 genes positively correlated with the expression of LSM5, LSM7 and LSM8 in GC. **(D)** The mRNA expression level of LSM5 was higher in the non-sensitive group compared to sensitive group. Groups were selected by the effects of GC patients with 5-FU chemotherapy. 13 GC patients here had paired tumor and normal tissues. ns, p ≥ 0.05; *p < 0.05; **p < 0.01. **(E)** LSM7 had no significance betwwen two groups. **(F)** The mRNA expression level of LSM8 was higher in the non-sensitive group compared to sensitive group. All of above were from TCGA database.

We then identified differentially expressed genes (DEGs) in the high LSM5, 7 and 8 expression group in GC compared to the low expression group respectively (**Figures 5D–F**). The thirty most significant DEGs in GC were shown in the single gene co-expression heat map (**Figures 6A–C**). Most importantly, to elucidate the certain role of LSM5, 7 and 8, we conducted qPCR in 13 GC patients that had paired tumor and normal tissues. These patients were divided into two groups according to the curative efficiency of neoadjuvant chemotherapy, including sensitive and nonsensitive. The results showed that the mRNA expression level of LSM5 and LSM8 was lower in the sensitive group compared to non-sensitive group while LSM7 showed no significance (**Figures 6D–F**), which suggested that LSM5 and LSM8 may promote 5-FU chemoresistance in GC.

### 3.7 The features of immune infiltration, MSI statues and TMB for LSM5 and LSM8 in GC patients

**Figure 7:**
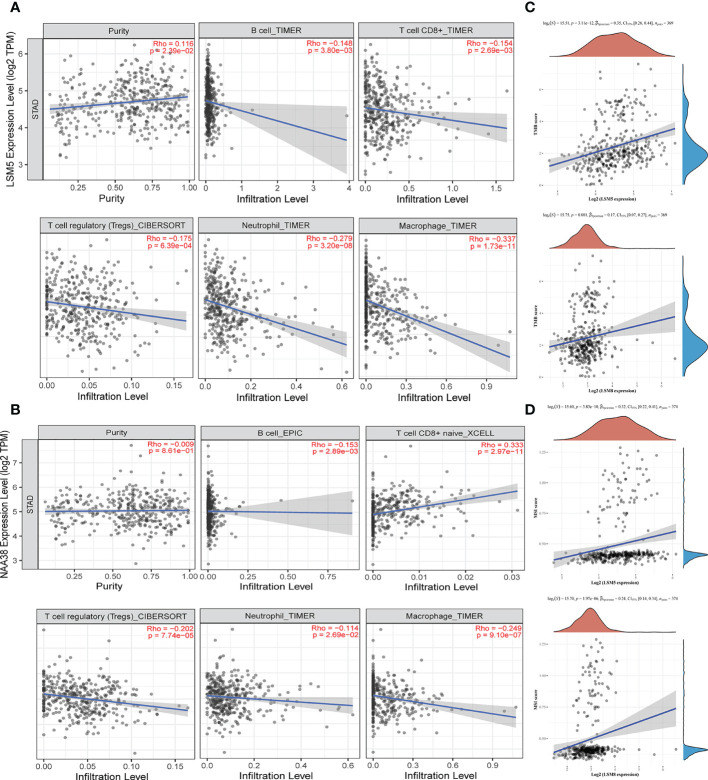
The status of tumor microenvironment for LSM5 and LSM8 in GC. **(A, B)** Correlations between the abundance of immune cells and the expression of LSM5, LSM8 by TIMER analysis (https://cistrome.shinyapps.io/timer/). **(C)** The expression of LSM5 and LSM8 positively correlated with TMB in GC from TCGA database. **(D)** The expression of LSM5 and LSM8 positively correlated with MSI in GC from TCGA database.

Tumor microenvironment (TME) is associated with relapse and chemoresistance of cancer cell. In this study, the correlation between immune cell infiltration, microsatellite instability (MSI) statues, tumor mutational burden (TMB) with LSM members were explored, and due to the above validations, we mainly focused on LSM5 and LSM8. The expression of LSM5 was in negative correlation with the infiltration of B cells, CD8+ T cells, Tregs, neutrophils and macrophages in GC patients (**Figure 7A**). However, LSM8 was positively associated with the infiltration of CD8+ T cells and negatively associated with the infiltration of B cells, Tregs, neutrophils and macrophages in GC patients (**Figure 7B**). With regard to the rest LSM members, multiple immune cells have correlation in GC patients, including CD4+ T cells, neutrophils, B cells, CD8+ T cells, macrophages, and dendritic cells (**Figure S4**). Then we found that all LSM family members were positively associated with TMB in GC patients (**Figures 7C**; **S5**). Also, most members of LSM family were positively related to the statues of MSI in GC patients, excepting LSM2 and LSM10 (**Figures 7D**; **S6**).

### 3.8 The association of the increased expression of LSM5 and LSM8 with the decreased expression level of immune cell infiltration

**Figure 8:**
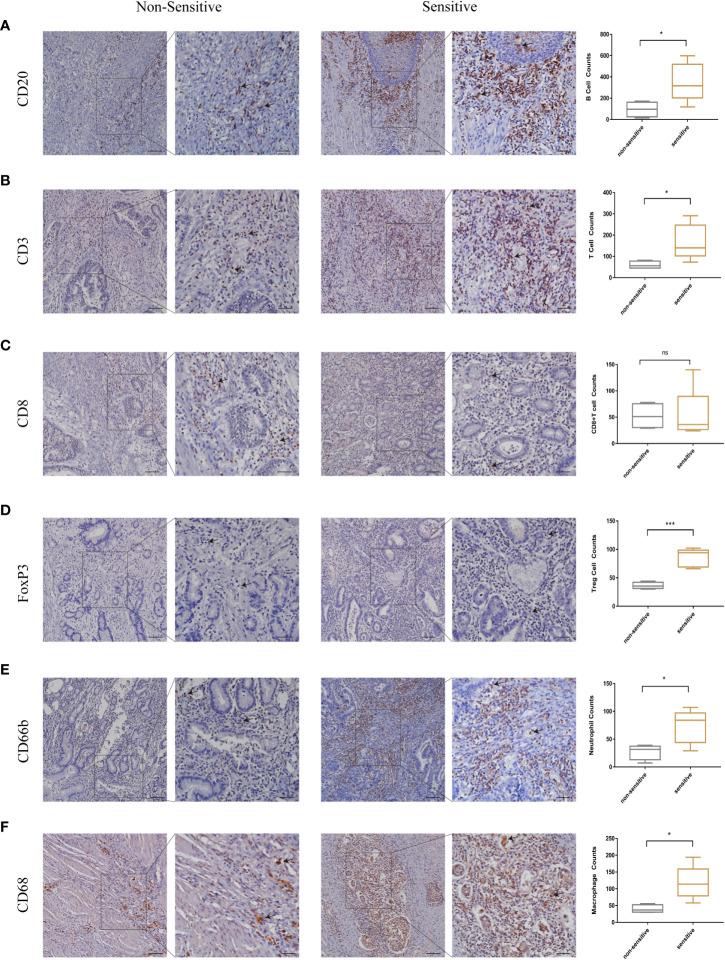
The validation of immune infiltration in clinical GC tissues. **(A, B)** the infiltration of B cells and T cells were higher in the sensitive group compared to non-sensitive group. **(C)** the expression of CD8+T cells showed no differences between the two groups. **(D–F)** higher expression levels of Tregs, neutrophils and macrophages were found in the sensitive group compared to nonsensitive group. Data were shown as mean ± SD. ns, p ≥ 0.05; *p < 0.05; ***p < 0.001.

In this study, we have firstly identified LSM5 and LSM8 as favourable biomarkers related to 5-FU chemotherapy resistance and investigated their relationship with tumor microenvironment and immune therapy in GC patients. Furthermore, to determine the relationship between immune cell infiltration and chemotherapy effect in higher level of LSM5 and LSM8 patients, we verified the immune infiltration level of B cells, CD8+ T cells, Tregs, neutrophils and macrophages in GC patients selected for qPCR experiment. Finally, 4 GC patients in the non-sensitive group and 5 cases in the sensitive group were included for IHC test for expression of LSM5 and LSM8. As shown in **Figure 8**, the infiltration of CD8+T cells showed no differences between the two groups. However, higher expression levels of B cells, T cells, Tregs, neutrophils and macrophages were found in the sensitive group compared to non-sensitive group, which was highly consistent with the results of TIMER database.

### 3.9 Functional enrichment analysis of LSM5 and LSM8 associated differentially expressed genes in gastric cancer

**Figure 9:**
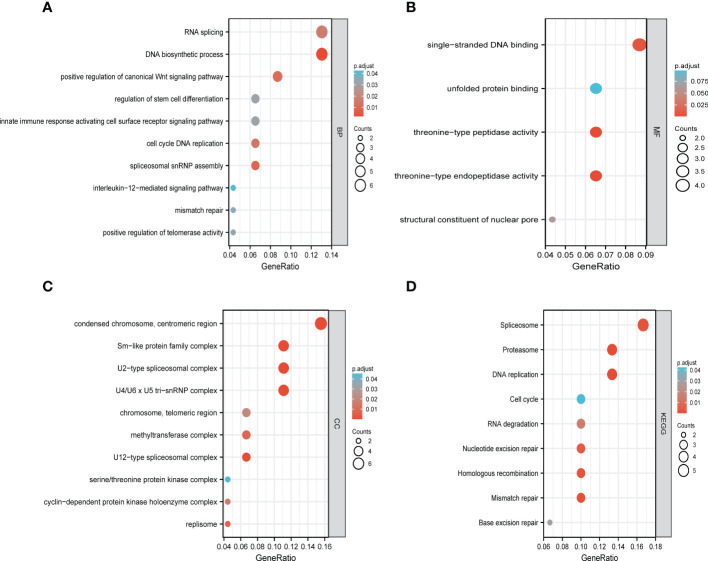
Functional enrichment analysis of DEGs based on 50 targeted binding proteins of LSM5 and LSM8 in GC. **(A-C)** GO enrichment analysis of the LSM5 and LSM8-associated DEGs show the enriched biological functions (BP), cellular components (CC), and molecular functions (MF). **(D)** KEGG analysis of the LSM5 and LSM8-associated DEGs. Above were analyzed by GEPIA 2database (http://gepia2.cancer-pku.cn/#in http://gepia2.cancer-pku.cn/#indexdex).

The functions of LSMs and the genes significantly associated with LSM alterations were predicted by analyzing gene ontology (GO) and Kyoto Encyclopedia of Genes and Genomes (KEGG). GO enrichment analysis predicted the functional roles of target host genes based on three aspects. In GC, we found that RNA splicing, DNA biosynthetic process, positive regulation of canonical Wnt signalling pathway, regulation of stem cell differentiation and innate immune response activating cell surface receptor signalling pathway were significantly regulated by LSM alterations on biological processes (**Figure 9A**). In addition, condensed chromosome, centromeric region, Sm-like protein family complex and U2-type spliceosomal complex on cellular components (**Figure 9B**), singlestranded DNA binding, unfolded protein binding and threonine-type peptidase activity on molecular functions (**Figure 9C**) were also significantly controlled by these LSM alterations. KEGG analysis defined the pathways related to the functions of LSM alterations and the frequently altered neighbour genes. Totally 9 pathways related to the functions of LSM alterations in GC were found through KEGG analysis (**Figure 9D**), containing spliceosome, proteasome, DNA replication, cell cycle, RNA degradation, nucleotide excision repair, homologous recombination, mismatch repair and base excision repair.

## 4 Discussion

Although nearly 50% of advanced GC can undergo radical resection, the recurrence rate remains obstinately high (18). Both MAGIC study and FNCLCC/FFCD trial proved that perioperative chemotherapy combined with surgical resection was superior to simple surgery and thus prolonged life survival, which established the key role of chemotherapy for GC (19, 20). However, clinical drug resistance become the most obstacle of chemotherapy, resulting poor prognosis of GC. So it is urgent to find novel potential biomarkers for predicting and improving chemotherapy sensitivity. In this study, we found that all members of LSM family were highly expressed in GC tissues compared with normal gastric tissues, which suggested that it may be related to the progression of GC. Consistently, other studies revealed LSM1, LSM2, LSM3, LSM12 as oncogenes which could play a crucial role in growth and progress of breast cancer (7), basal-like primary tumors (21), cervical carcinoma (22) or colorectal cancer (23). Furthermore, genetic alterations analysis of LSM family genes showed that amplification alterations of LSM family were more common in GC and it may correlate with the progression of tumor based on previous researches. Noblejas et al. revealed that amplification of the LSM1 gene in luminal breast cancer was significantly related with poor clinical outcome (24).

Intriguingly, LSM2, LSM4, LSM5, LSM6, LSM7, LSM8, LSM12 and LSM14B showed relatively high accuracy (AUC > 0.8) in predicting the prognosis of patients with GC. Previous studies also revealed LSM2 as an independent predictor of poor prognosis in ovarian cancer (25). Then, we paid much attention to the prognostic value of LSM family in GC patients with 5-FU chemotherapy. High expression levels of LSM2, LSM3, LSM4, LSM5, LSM6, LSM7, LSM8, LSM11, and LSM14A were associated with shorter OS of GC patients undergoing 5-FU based chemotherapy, while LSM12 was on the contrary.

Moreover, these results reflected that LSM members were involved in estimating 5-FU therapy effects in general, but we were curious that which LSM member played the key role and whether it changed through the whole process of 5-FU treatment. Firstly, we processed GEO2R in GSE14210 to compare pretreatment and posttreatment endoscopic biopsy samples collected from cisplatin and fluorouracil combination chemotherapy in GC patients. We found that LSM5, LSM7 and LSM8, as hub genes, upregulated mostly after 5-FU treatment compared to other LSM members, which indicated the high correlation with 5-FU chemoresistance in GC. Furthermore, we conducted qPCR experiments at matched tissues in GC patients with neoadjuvant chemotherapy who were divided into two groups according to the degree of neoadjuvant chemotherapy sensitivity. The results indicated the mRNA level of LSM5 and LSM8 was higher in non-sensitive group compared to that of sensitive group, while LSM7 showed no significance, which suggested that overexpression of LSM5 and LSM8 may promote 5-FU chemoresistance in GC.

Previous studies mostly explored the function of LSM5 and LSM8 in arabidopsis (26, 27). However, there is lack of studies on the role of LSM5 and LSM8 in tumors. Recent study revealed a risk score system consisting of LSM5 and identified it as a reliable predictive biomarker for hepatocellular cancer (28). So it is challenging but greatly significant to explore the underlying molecular mechanism about why LSM5 and LSM8 could be potential biomarkers for chemoresistant GC patients, we firstly analyzed the GO enrichment and KEGG annotation in GC. The results showed that DNA replication, RNA splicing, spliceosome, proteasome and Sm-like protein family complex may participate in this procedure, which was consistent with the following studies. Hongkai, et al. demonstrated that a different hetero-heptameric complex of LSM proteins (LSM2-8) affected the processing of pre-mRNA and small stable RNAs in the nucleus (29). In addition, Naimur et al. found that the nuclear LSM2-8 complex was pro-viral and knockdown of LSM8 reduced RNA levels of hepatitis B virus (30). To date, the nuclear LSm2-8 are one of the best characterized complexes in eukaryotes. Therefore,Combined with the topmost Pearson coefficient between LSM5 and 8, we preliminarily proposed that there may be the existence of LSM5 and LSM8 complex that played a role in GC chemoresistance.We then investigated KEGG analysis of LSM5, LSM8 and related genes and found that positive regulation of canonical Wnt signaling pathway and regulation of stem cell differentiation were significant. As we all know, the Wnt pathway participates in cell proliferation, cell polarity and cell fate determination (31). Also, cancer stem cells(CSCs) possessed inherent resistance to chemotherapy through the Wnt pathway (32). Based on the above analysis, we proposed a hypothesis that the interaction of Wnt signaling pathway and regulation of stem cell differentiation may be regulated by LSM5 and LSM8 complex, which suggested that LSM5 and LSM8 could be potential biomarkers for chemoresistant GC patients in molecular biological level. And the verification of molecular mechanism was planned in the next research to provide a more solid theoretical basis and clinical transformation for LSM5 and LSM8 in GC chemoresistance.

In addition, we investigated the relationship of immune infiltration with LSM5 and LSM8. Because chemotherapy combined with immunotherapy have drawn more and more attention to improve the therapeutic effect for GC recently. Qiao et al. found that Dendritic Cell-Cytokine Induced Killer combined with S-1 plus cisplatin provided a favorable PFS and OS in patients with AGC, and the combination therapy was safe with tolerable toxicities (33). More enlightening was that the long-term success of traditional chemotherapeutics and targeted anticancer agents mostly depends on immunological effects in Galluzzi’s study (34). Our study proved that both LSM5 and LSM8 were in negative connection with the infiltration of B cells, Tregs, neutrophils and macrophages in GC patients, whereas infiltration of CD8+ T cells was contrary. As we all know, immune cells can be divided into immune effector cells and suppressor cells according to their functions. For example, Tregs are immunosuppresive and generally downregulate induction and proliferation of effector T cells. Furthermore, the IHC results of GC patients proved that a higher expression of B cells, T cells, Tregs, neutrophils and macrophages was revealed in the sensitive group where the expression level of LSM5 and 8 was relative lower, whereas the infiltration of CD8+T cells showed no differences in two groups. It directly revealed the relationship between the expression of LSM5 or LSM8 with the immune microenvironment in the chemotherapy resistant group. Consistent with previous studies, robust tumor infiltrations by B cells, T cells, tumor associated macrophages, Treg cells were proved to be correlated with improved OS in biliary tract cancer (35), rectal carcinoma (36), gastric cancer (37) with adjuvant chemotherapy. Interestingly, the infiltration of both immune effector cells and suppressor cells in this study were generally low in non-sensitive tissues. This suggested that patients resistant to chemotherapy may not be sensitive to immunotherapy either, and we should find out the reason for low expression of immune effector cells. Obviously, immune effector cells weren’t directly regulated by the suppressor cells here and we supposed that this may be attributed to the genes or intrinsic features of GC. In a word, it provided new perspectives to chemotherapy combined with immunotherapy in GC and laid a foundation for future research.

To our knowledge, we were the first to comprehensively investigate the role of LSM family members in GC and identify LSM5 and LSM8 as potential biomarkers for GC with chemotherapy resistance through screening of data analysis and experiments. We still have many limitations and many efforts to improve it. Firstly, there are relatively small samples and we need a larger-scaled sample and study. Secondly, it’s necessary for us to conduct more basic research exploration, including cell and animal experiments. Finally, in order to be more practical and persuasive in clinical transformation, we need to explore the mechanism of why LSM5 and LSM8 can be biomarker of chemoresistance in GC, explaining it from a deep perspective and further exploring the way to achieve clinical intervention. For this research, we are willing to demonstrate and elucidate it in our future study.

## Data availability statement

The original contributions presented in the study are included in the article/**Supplementary Material**. Further inquiries can be directed to the corresponding author.

## Ethics statement

The studies involving human participants were reviewed and approved by the Ethics Committee of Third Affiliated Hospital of Sun Yat-Sun University ([2022]02-169-01). The patients/participants provided their written informed consent to participate in this study. Written informed consent was obtained from the individual(s) for the publication of any potentially identifiable images or data included in this article.

## Author contributions

The authors confirm contribution to the paper as follows: Conceptualization, QLiu, CJ and JF; Data curation, SY; Formal analysis, QLian; Funding acquisition, CJ and JF; Methodology, CJ; Project administration, CJ and JF; Resources, SY; Software, QLiu and QLian; Supervision, JF; Validation, QLiu and YS; Visualization, QLiu and QLian; Writing – original draft, QLiu, QLian, CJ and JF; Writing – review & editing, QLiu, QLian, CJ and JF. All authors contributed to the article and approved the submitted version.
