# Supplementary material for: Identification of LSM family members as potential chemoresistance predictive and therapeutic biomarkers for gastric cancer
Source: Front Oncol. 2023 Mar 17;13:1119945. doi: 10.3389/fonc.2023.1119945 (PMC10064066; doi:10.3389/fonc.2023.1119945)
Supplement: Supplementary file 1 [file DataSheet_1.pdf]

Supplementary Material

Identification of LSM Family Members as Novel Chemoresistance Predictive and Therapeutic Biomarkers for Gastric Cancer

Qianhui Liu[1†], Qinghai Lian[2†], Yingqiu Song[1], Shangbin Yang[1], Changchang Jia[2*], and Jiafeng Fang[1*]

*** Correspondence:** Jiafeng Fang* fangjf@mail.sysu.edu.cn

Supplementary Material

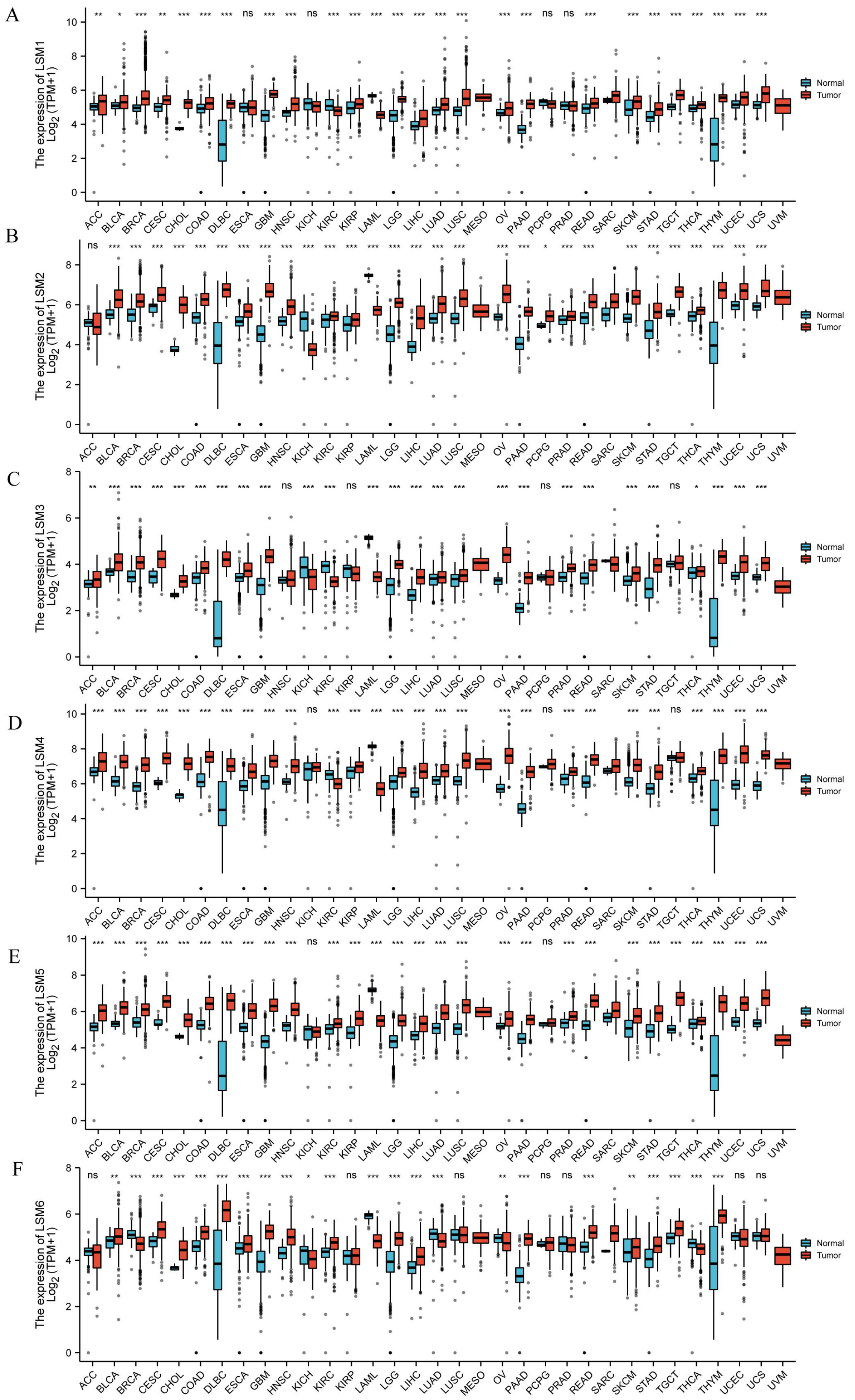
A
The expression of LSM1
Log2 (TPM+1)
B
The expression of LSM2
Log2 (TPM+1)
C
The expression of LSM3
Log2 (TPM+1)
D
The expression of LSM4
Log2 (TPM+1)
E
The expression of LSM5
Log2 (TPM+1)
F
The expression of LSM6
Log2 (TPM+1)
ACC BLCA BRCA CESC CHOL COAD DLBC ESCA GBM HNSC KICH KIRC KIRP LAML LGG LIHC LUAD LUSC MESO OV PAAD PCPG PRAD READ SARC SKCM STAD TGCT THCA THYM UCEC UCS UVM
Normal
Tumor

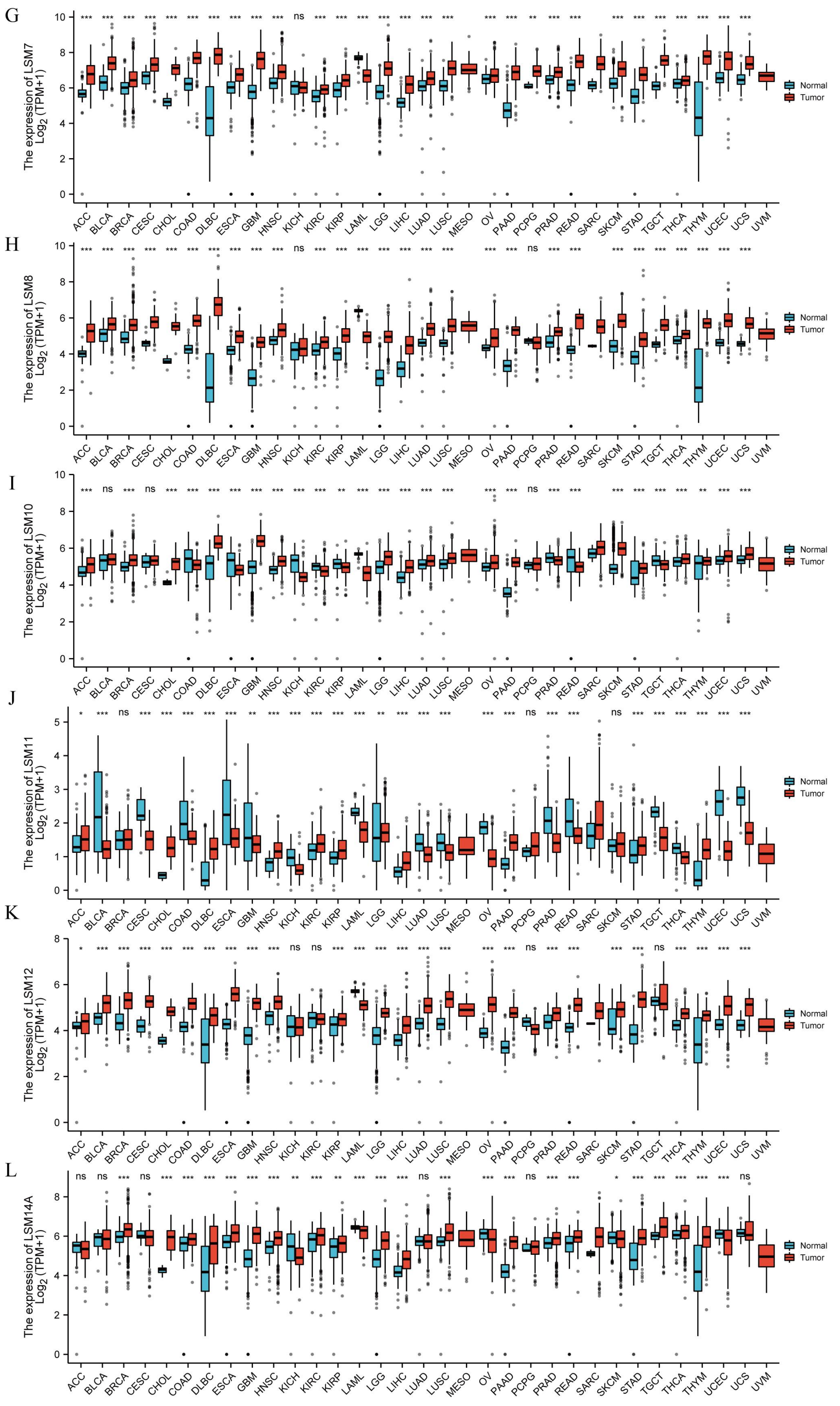

G
The expression of LSM7
Log2 (TPM+1)
H
The expression of LSM8
Log2 (TPM+1)
I
The expression of LSM10
Log2 (TPM+1)
J
The expression of LSM11
Log2 (TPM+1)
K
The expression of LSM12
Log2 (TPM+1)
L
The expression of LSM14A
Log2 (TPM+1)
Normal
Tumor
ACC BLCA BRCA CESC CHOL COAD DLBC ESCA GBM HNSC KICH KIRC KIRP LAML LGG LIHC LUAD LUSC MESO OV PAAD PCPG PRAD READ SARC SKCM STAD TGCT THCA THYM UCEC UCS UVM

M

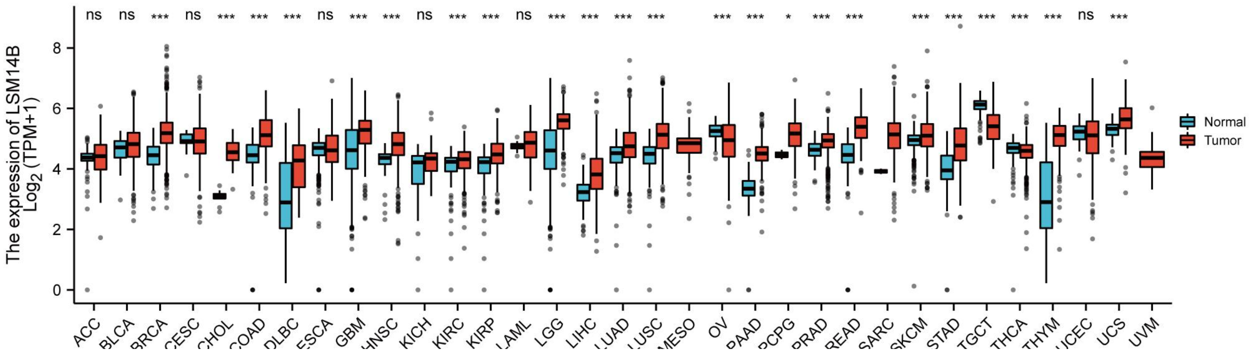

Supplementary Figure 1. Expression level of 13 LSM members in 33 types of cancer tissues and their corresponding normal tissues.

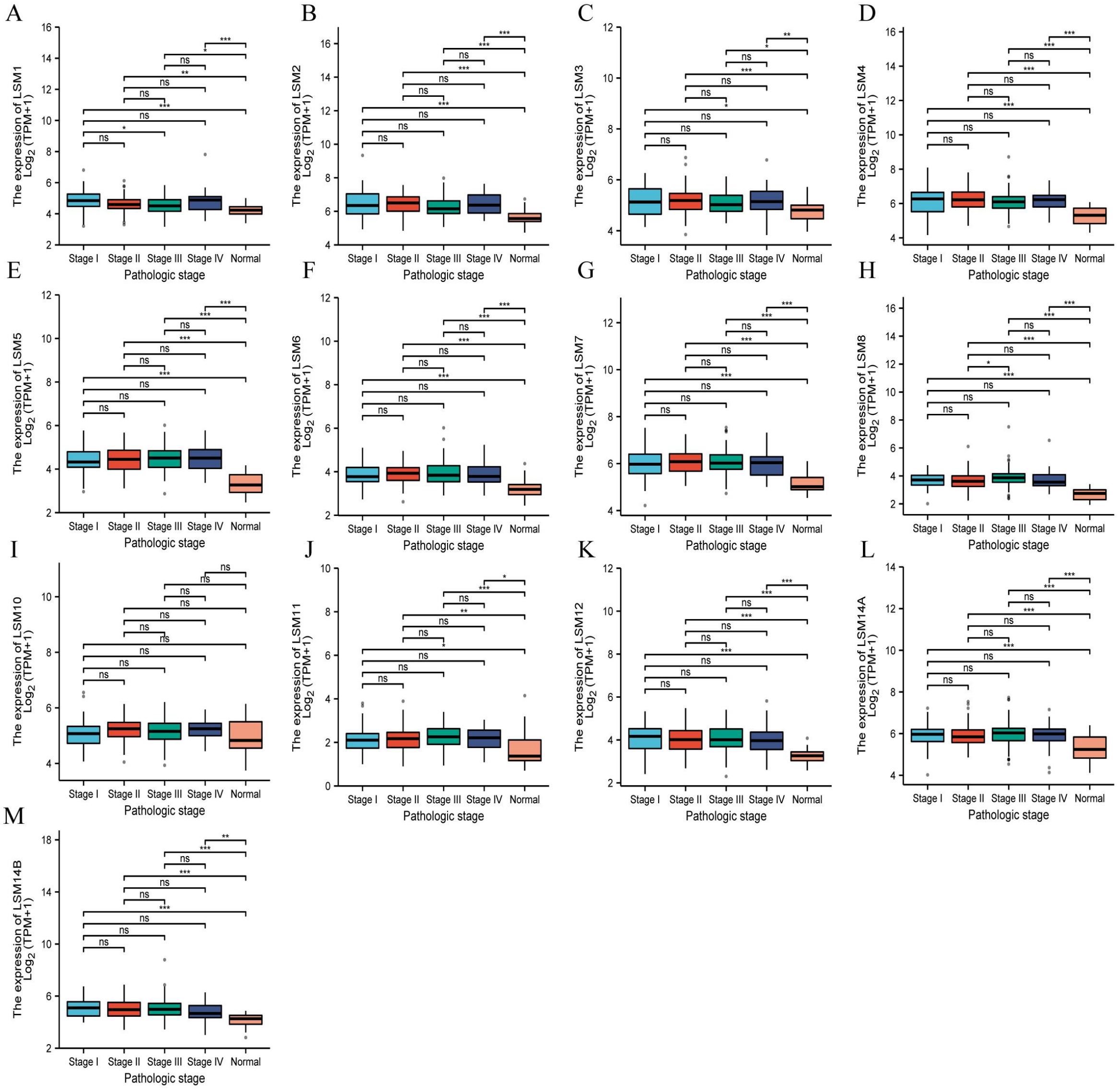

Supplementary Figure 2. Relationship between the mRNA expression of LSM genes and individual pathological stage of GC patients.

A B

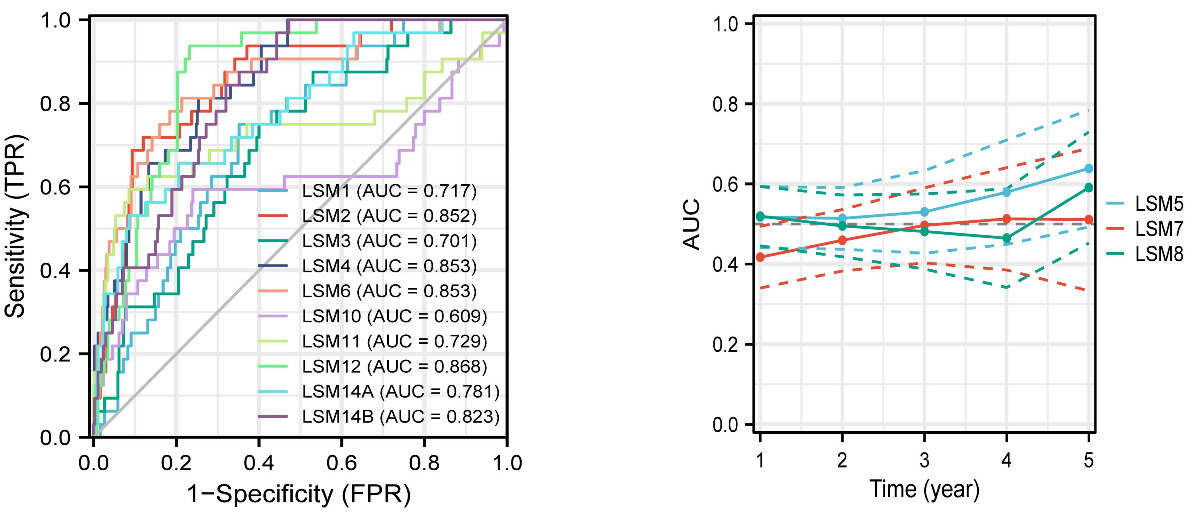

Supplementary Figure 3. A. Receiver operating characteristic (ROC) curve for LSM family members expression except LSM5, LSM7 and LSM8 in GC. B. Time-ROC curve for LSM5, LSM7 and LSM8 in GC.

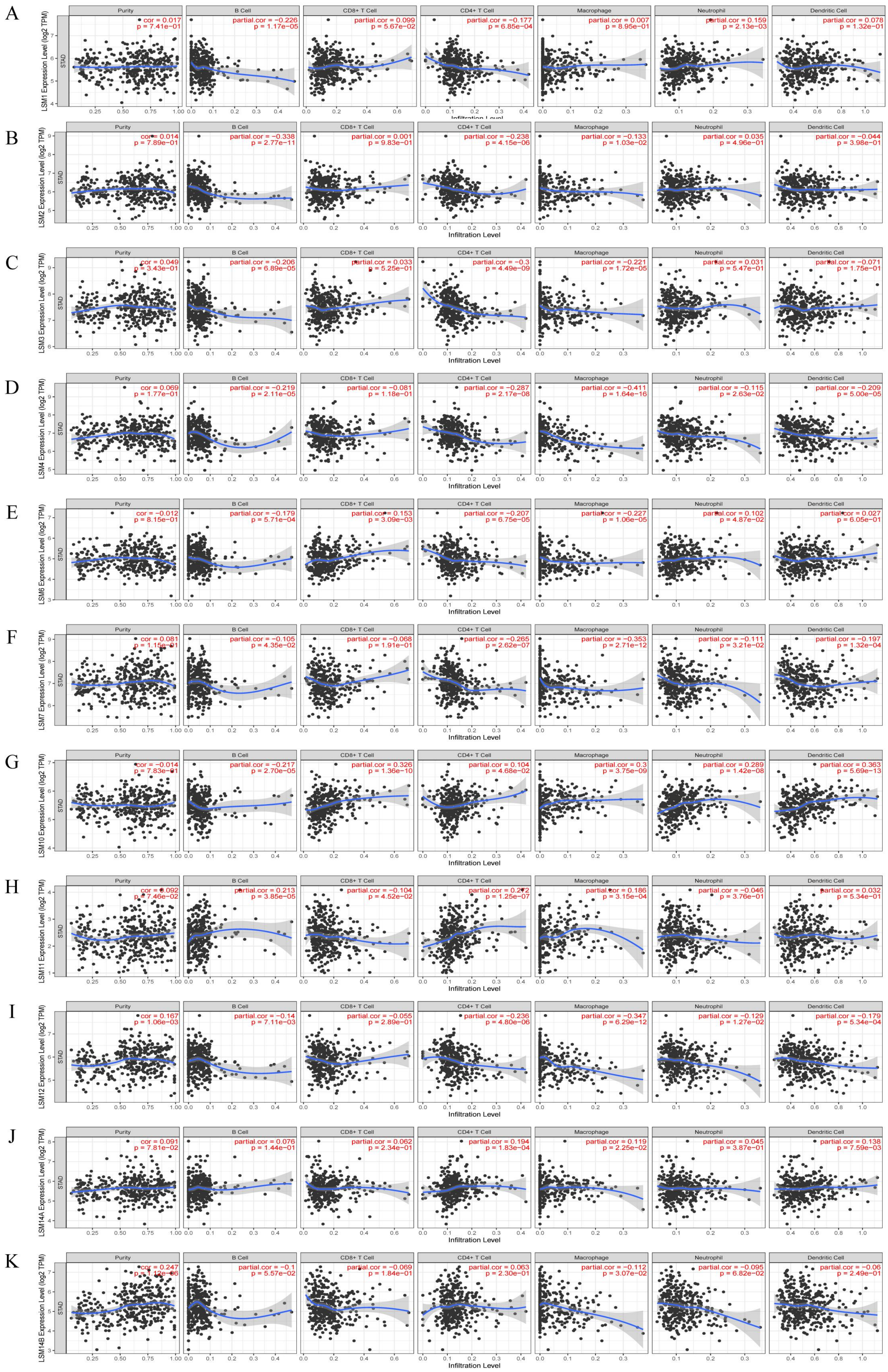

A
LSM1 Expression Level (log2 TPM)
STAD
Purity cor = 0.017 p = 7.41e−01
B Cell partial.cor = −0.226 p = 1.17e−05
CD8+ T Cell partial.cor = 0.099 p = 5.67e−02
CD4+ T Cell partial.cor = −0.177 p = 6.85e−04
Macrophage partial.cor = 0.007 p = 8.95e−01
Neutrophil partial.cor = 0.159 p = 2.13e−03
Dendritic Cell partial.cor = 0.078 p = 1.32e−01
B
LSM2 Expression Level (log2 TPM)
STAD
Purity cor = 0.014 p = 7.89e−01
B Cell partial.cor = −0.338 p = 2.77e−11
CD8+ T Cell partial.cor = 0.001 p = 9.83e−01
CD4+ T Cell partial.cor = −0.238 p = 4.15e−06
Macrophage partial.cor = −0.133 p = 1.03e−02
Neutrophil partial.cor = 0.035 p = 4.96e−01
Dendritic Cell partial.cor = −0.044 p = 3.98e−01
Infiltration Level
C
LSM3 Expression Level (log2 TPM)
STAD
Purity cor = 0.049 p = 3.43e−01
B Cell partial.cor = −0.206 p = 6.89e−05
CD8+ T Cell partial.cor = 0.033 p = 5.25e−01
CD4+ T Cell partial.cor = −0.3 p = 4.49e−09
Macrophage partial.cor = −0.221 p = 1.72e−05
Neutrophil partial.cor = 0.031 p = 5.47e−01
Dendritic Cell partial.cor = −0.071 p = 1.75e−01
Infiltration Level
D
LSM4 Expression Level (log2 TPM)
STAD
Purity cor = 0.069 p = 1.77e−01
B Cell partial.cor = −0.219 p = 2.11e−05
CD8+ T Cell partial.cor = −0.081 p = 1.18e−01
CD4+ T Cell partial.cor = −0.287 p = 2.17e−08
Macrophage partial.cor = −0.411 p = 1.64e−16
Neutrophil partial.cor = −0.115 p = 2.63e−02
Dendritic Cell partial.cor = −0.209 p = 5.00e−05
Infiltration Level
E
LSM6 Expression Level (log2 TPM)
STAD
Purity cor = −0.012 p = 8.15e−01
B Cell partial.cor = −0.179 p = 5.71e−04
CD8+ T Cell partial.cor = 0.153 p = 3.09e−03
CD4+ T Cell partial.cor = −0.207 p = 6.75e−05
Macrophage partial.cor = −0.227 p = 1.06e−05
Neutrophil partial.cor = 0.102 p = 4.87e−02
Dendritic Cell partial.cor = 0.027 p = 6.05e−01
Infiltration Level
F
LSM7 Expression Level (log2 TPM)
STAD
Purity cor = 0.081 p = 1.15e−01
B Cell partial.cor = −0.105 p = 4.35e−02
CD8+ T Cell partial.cor = −0.068 p = 1.91e−01
CD4+ T Cell partial.cor = −0.265 p = 2.62e−07
Macrophage partial.cor = −0.353 p = 2.71e−12
Neutrophil partial.cor = −0.111 p = 3.21e−02
Dendritic Cell partial.cor = −0.197 p = 1.32e−04
Infiltration Level
G
LSM10 Expression Level (log2 TPM)
STAD
Purity cor = −0.014 p = 7.83e−01
B Cell partial.cor = −0.217 p = 2.70e−05
CD8+ T Cell partial.cor = 0.326 p = 1.36e−10
CD4+ T Cell partial.cor = 0.104 p = 4.68e−02
Macrophage partial.cor = 0.3 p = 3.75e−09
Neutrophil partial.cor = 0.289 p = 1.42e−08
Dendritic Cell partial.cor = 0.363 p = 5.69e−13
Infiltration Level
H
LSM11 Expression Level (log2 TPM)
STAD
Purity cor = 0.092 p = 7.46e−02
B Cell partial.cor = 0.213 p = 3.85e−05
CD8+ T Cell partial.cor = −0.104 p = 4.52e−02
CD4+ T Cell partial.cor = 0.272 p = 1.25e−07
Macrophage partial.cor = 0.186 p = 3.15e−04
Neutrophil partial.cor = −0.046 p = 3.76e−01
Dendritic Cell partial.cor = 0.032 p = 5.34e−01
Infiltration Level
I
LSM12 Expression Level (log2 TPM)
STAD
Purity cor = 0.167 p = 1.06e−03
B Cell partial.cor = −0.14 p = 7.11e−03
CD8+ T Cell partial.cor = −0.055 p = 2.89e−01
CD4+ T Cell partial.cor = −0.236 p = 4.80e−06
Macrophage partial.cor = −0.347 p = 6.29e−12
Neutrophil partial.cor = −0.129 p = 1.27e−02
Dendritic Cell partial.cor = −0.179 p = 5.34e−04
Infiltration Level
J
LSM14A Expression Level (log2 TPM)
STAD
Purity cor = 0.091 p = 7.81e−02
B Cell partial.cor = 0.076 p = 1.44e−01
CD8+ T Cell partial.cor = 0.062 p = 2.34e−01
CD4+ T Cell partial.cor = 0.194 p = 1.83e−04
Macrophage partial.cor = 0.119 p = 2.25e−02
Neutrophil partial.cor = 0.045 p = 3.87e−01
Dendritic Cell partial.cor = 0.138 p = 7.59e−03
Infiltration Level
K
LSM14B Expression Level (log2 TPM)
STAD
Purity cor = 0.247 p = 1.12e−06
B Cell partial.cor = −0.1 p = 5.57e−02
CD8+ T Cell partial.cor = −0.069 p = 1.84e−01
CD4+ T Cell partial.cor = 0.063 p = 2.30e−01
Macrophage partial.cor = −0.112 p = 3.07e−02
Neutrophil partial.cor = −0.095 p = 6.82e−02
Dendritic Cell partial.cor = −0.06 p = 2.49e−01
Infiltration Level

Supplementary Figure 4. Immune infiltration levels for LSM family members expression except LSM5 and LSM8 in GC.

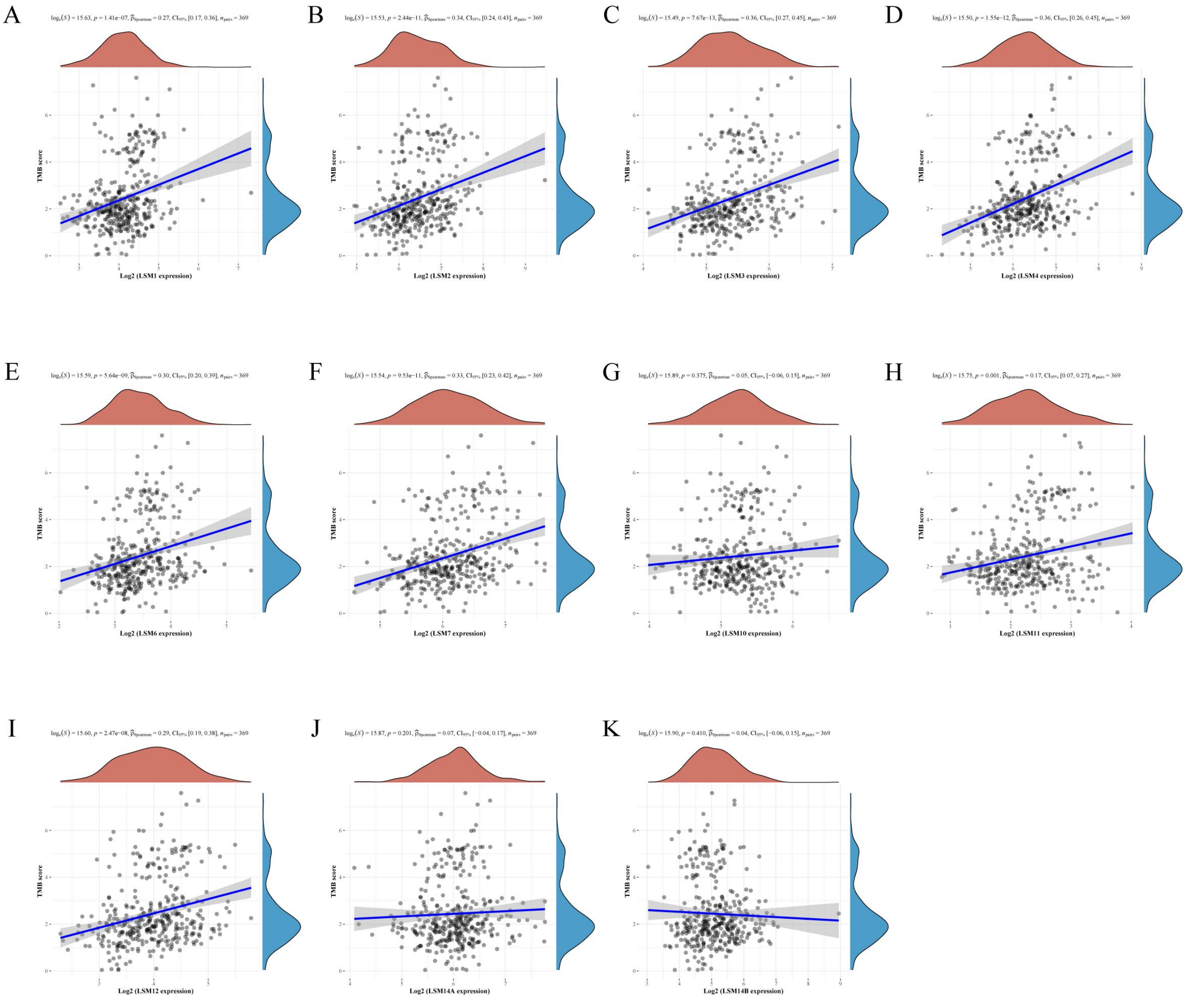

Supplementary Figure 5. Correlations between the TMB score and the expression of LSM family members expression except LSM5 and LSM8 in GC.

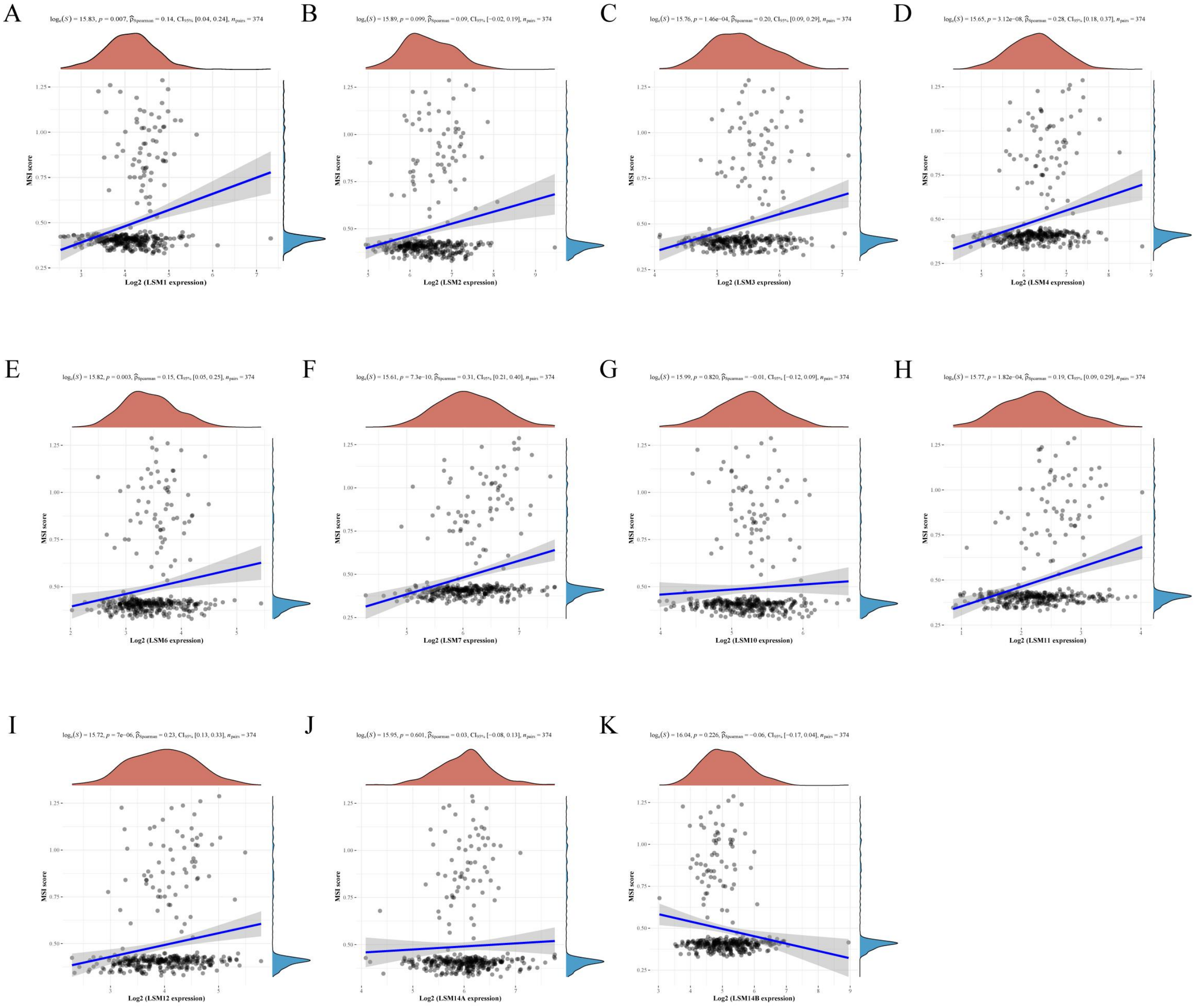

Supplementary Figure 6. Correlations between the MSI score and the expression of LSM family members expression except LSM5 and LSM8 in GC.
